# Antiviral Activity and Crystal Structures of HIV-1 gp120 Antagonists

**DOI:** 10.3390/ijms232415999

**Published:** 2022-12-15

**Authors:** Francesca Curreli, Young D. Kwon, Isabella Nicolau, Giancarla Burgos, Andrea Altieri, Alexander V. Kurkin, Raffaello Verardi, Peter D. Kwong, Asim K. Debnath

**Affiliations:** 1 Laboratory of Molecular Modeling and Drug Design, Lindsey F. Kimball Research Institute, New York Blood Center, New York, NY 10065, USA; 2 Vaccine Research Center, National Institute of Allergy and Infectious Diseases, National Institutes of Health, Bethesda, MD 20892, USA; 3 EDASA Scientific srls, Via Stingi 37, 66050 San Salvo, Italy

**Keywords:** HIV-1, envelope glycoprotein (Env), gp120 antagonist, crystal structure, antiviral, cytotoxicity

## Abstract

As part of our effort to discover drugs that target HIV-1 entry, we report the antiviral activity and crystal structures of two novel inhibitors in a complex with a gp120 core. NBD-14204 showed similar antiviral activity against all the clinical isolates tested. The IC_50_ values were in the range of 0.24–0.9 µM with an overall mean of 0.47 ± 0.03 µM, showing slightly better activity against the clinical isolates than against the lab-adapted HIV-1_HXB2_ (IC_50_ = 0.96 ± 0.1 µM)_._ Moreover, the antiviral activity of NBD-14208 was less consistent, showing a wider range of IC_50_ values (0.66–5.7 µM) with an overall mean of 3 ± 0.25 µM and better activity against subtypes B and D (Mean IC_50_ 2.2–2.5 µM) than the A, C and Rec viruses (Mean IC_50_ 2.9–3.9 µM). SI of NBD-14204 was about 10-fold higher than NBD-14208, making it a better lead compound for further optimization. In addition, we tested these compounds against S375Y and S375H mutants of gp120, which occurred in some clades and observed these to be sensitive to NBD-14204 and NBD-14208. These inhibitors also showed modest activity against HIV-1 reverse transcriptase. Furthermore, we determined the crystal structures of both inhibitors in complexes with gp120 cores. As expected, both NBD-14204 and NBD-14208 bind primarily within the Phe43 cavity. It is noteworthy that the electron density of the thiazole ring in both structures was poorly defined due to the flexibility of this scaffold, suggesting that these compounds maintain substantial entropy, even when bound to the Phe43 cavity.

## 1. Introduction

Advances in available therapeutics, particularly combination antiretroviral therapy, have significantly improved the treatment of HIV infections, facilitating the shift from a disease characterized by high morbidity and mortality to a manageable chronic disease. Despite this remarkable success, current treatments suffer from several limitations, including (1) reliance on daily adherence, (2) long-term use resulting in long-term toxicity, (3) limited treatment options due to the development of drug resistance, (4) high costs, and (5) the failure of current treatments to eradicate HIV. In addition, despite tremendous efforts and investment, no effective vaccine or microbicide has been developed, and significant hurdles must be overcome to achieve an effective cure. Thus, the continued development of small-molecule drugs with high potency against novel targets and minimal side effects remains imperative. The development of novel therapeutics will increase the number of available drugs, extend the scope of combination therapies, and present opportunities to formulate long-acting drugs.

Approximately 18 years after the first publication describing HIV-1 attachment inhibitors by Bristol Meyer & Squib (BMS) [1], the first drug targeting gp120, Fostemsavir [2] (Rukobia, Viiv Healthcare, Middlesex, TW8 9GS, UK), received FDA approval in 2021. This recognition by the FDA confirms the significance of our efforts to develop novel drugs targeting HIV-1 gp120, especially for the growing number of treatment-experienced patients with limited treatment options and for use in combination therapies for the millions of affected individuals worldwide.

Our group has made significant progress toward meeting this critical need by developing a new class of HIV-1 entry inhibitors (we termed NBDs) targeting the Phe43 cavity of HIV-1 gp120, a different binding site from that targeted by Fostemsavir. NBDs inhibit gp120 binding to the cellular receptor CD4 by direct binding to the Phe43 cavity of HIV-1 envelope glycoprotein gp120. On the contrary, Fostemsavir binds to an induced binding pocket under the β20–21 loop, distinct from the Ph43 cavity created upon CD4 binding. We reported the design, synthesis, antiviral activity, X-ray crystal structures, and preclinical assessments of many such NBDs [3,4,5,6,7,8,9,10,11,12,13,14,15,16]. Due to the critical role the structural information plays in designing inhibitors with improved binding and antiviral activity, we continue to strive to determine the crystal structures of NBDs to understand the binding interactions of this series of compounds in the Phe43 cavity.

We recently discovered that some of the most active gp120 antagonists also display antiviral activity against HIV-1 reverse transcriptase (RT) [15]. HIV uses RT to convert its RNA into viral DNA in a process called reverse transcription, a post-entry process in the HIV-1 life cycle. Nucleoside reverse transcriptase inhibitors (NRTI) and non-nucleoside reverse transcriptase inhibitors (NNRTI) play a pivotal role in the highly active antiretroviral therapy (HAART) of AIDS patients.

**Figure 1:**
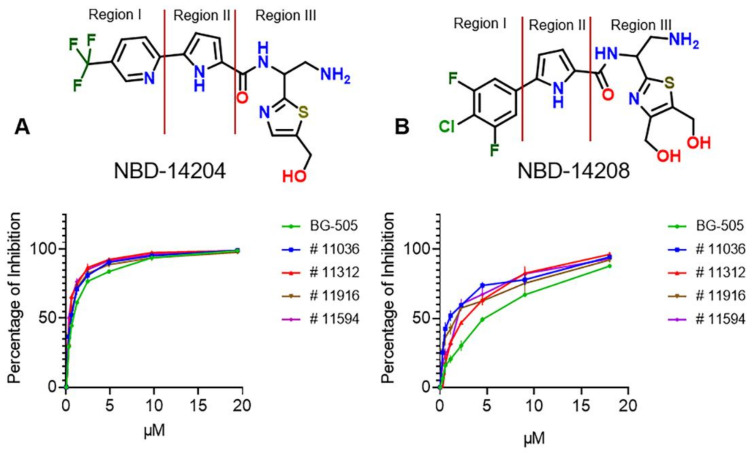
Chemical structures with region location and representative dose-response curves of NBD-14204 (**A**) and NBD-14208 (**B**) against five Env-pseudotyped clinical isolates.

This report showed detailed neutralizing activity of NBD-14204 (Figure 1A) and NBD-14208 (Figure 1B) against a selected set of HIV-1 pseudotyped viruses constructed with Env glycoproteins from a diverse class of clinical isolates. Earlier, we published the antiviral activity of these two NBDs against HIV-1_HXB2_ [12,13]. In addition, we presented the crystal structures of these compounds in complexes with gp120 cores.

## 2. Results & Discussion

### 2.1. Evaluation of the Antiviral Activity of NBD-14204 and NBD-14208 against Env-Pseudotyped Clinical Isolates

Our results have shown that compounds NBD-14204 and NBD-14208 have good antiviral activity against the laboratory-adapted Env-pseudotyped HIV-1_HXB-2_ [12,13]. Therefore, we decided to evaluate their activity against a selection of 25 Env-pseudotyped HIV-1 clinical isolate clones of different subtypes, including the A, B, C, and D types and five recombinant (Rec) HIV-1 clones. All those clinical isolates use CCR5 coreceptor for entry apart from subtype B # 11563, dual tropic (CCR5/CXCR4). As shown in Table 1, NBD-14204 showed equal antiviral activity against all the clinical isolates tested, including the Rec clones. The IC_50_ values were in the range of 0.24–0.9 µM with an overall mean of 0.47 ± 0.03 µM, showing slightly better activity against the clinical isolates than against the lab-adapted HIV-1_HXB2_ (IC_50_ = 0.96 ± 0.1 µM)_._ Representative dose-response curves are shown in Figure 1A. Moreover, the antiviral activity of NBD-14208 was less consistent, showing a wider range of IC_50_ values (0.66–5.7 µM) with an overall mean of 3 ± 0.25 µM and better activity against the subtypes B and D (Mean IC_50_ 2.2–2.5 µM) than the A, C and Rec viruses (Mean IC_50_ 2.9–3.9 µM). Representative curves are shown in Figure 1B. Additionally, both compounds were inactive against the control pseudovirus VSV-G suggesting their activity is specific to HIV-1.

### 2.2. S375Y and S375H Mutants Are Not Resistant to NBD-14204 and NBD-14208

We previously reported that some amino acid substitutions located in the CD4-binding site of gp120 reduced the sensitivity of those mutant viruses to the NBD compounds, but the sensitivities of viruses carrying the S375H, S375W, and S375Y mutations were not reduced [17]. Therefore, in this study, we evaluated both NBD compounds against two mutant pseudovirus HIV-1_HXB2_ having amino acid substitutions, S375Y and S375H, in the gp120 region. These mutants were sensitive to both NBD compounds, which showed similar activity against the mutants as was observed against HIV-1_HXB2_-WT (data not shown). S375 mutations, especially Y/N, are known to reduce sensitivity to HIV-1 attachment inhibitors [2,18]. In addition, a 48-week Phase 2b clinical trial reported several resistant mutants, including at S375, that reduced susceptibility to Fostemsavir [19].

### 2.3. NBD Compounds Showed Activity against HIV-1 Reverse Transcriptase (RT)

Some previously described NBD entry antagonists have shown activity against HIV-1 reverse transcriptase (RT) [11,15]. Therefore, we evaluated the RT inhibitory activity of NBD-14204 and NBD-14208 against that enzyme. We used Nevirapine and NBD-556 as controls. As expected, Nevirapine was highly potent against HIV-1 RT, with an IC_50_ of 0.20 μM. However, 300 μM NBD-556 had no activity (Table 2). Both compounds, NBD-14204 and NBD-14208, inhibited HIV-1 RT; their IC_50_s were 8.3 ± 1.2 μM and 5 ± 0.5 μM, respectively. Comparing those values with those obtained with the HIV-1 neutralization assays and calculating the ratios, we can hypothesize that the antiviral activity of NBD-14208 is primarily due to its activity against both gp120 and RT, while the primary activity of NBD-14204 is mostly against gp120 and its activity against RT may just play a secondary role. However, it was critically important to understand how some of the gp120 antagonists also show RT inhibitory activity. Since RTI and NNRTI drugs play a pivotal role in the highly active antiretroviral therapy (HAART) of AIDS patients, we wanted to learn more about the binding mode of these gp120 antagonists on HIV-1 RT. Therefore, we confirmed the binding of three such gp120 antagonists with HIV-1 RT by X-ray crystallography [15]. These compounds achieve dual-inhibitory activity by bridging dNTP- and NNRTI-binding sites to inhibit the polymerase activity of isolated RT in enzymatic assays. Similar binding was reported for compound G from Merck [20] (PDB: 5VZ6) and dihydroxybenzoyl naphthyl hydrazone [21] (DHBNH; PDB:2I5J). However, NBD binding extends into the NNRTI-binding pocket on one end and toward the polymerase active site and into the nucleotide-binding site on the other end, reaching further than either compound G or DHBNH1.

### 2.4. Crystal Structures of NBD-14204 and NBD-14208-Bound HIV-1 gp120 Core

**Figure 2:**
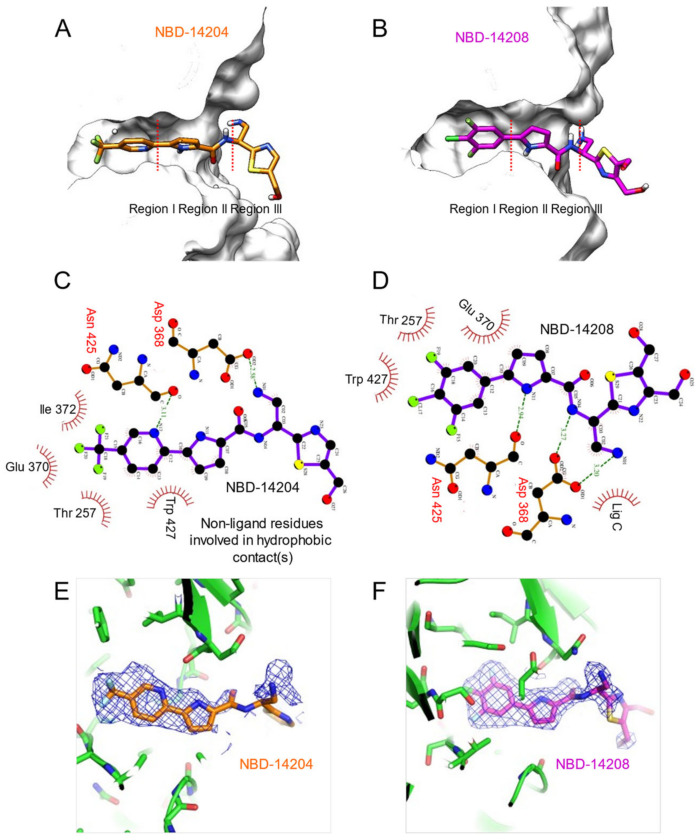
Crystal structures NBD-14204 and NBD-14208 in complex with gp120 core. (**A**) NBD-14204 in stick representation in the Phe43 cavity of gp120. (**B**) NBD-14208 in stick representation in the Phe43 cavity of gp120 (Regions I, II, and III are indicated in both structures. Detailed interactions of NBD-14204 (**C**) and NBD-14208 (**D**) with gp120. The dotted green lines represent the hydrogen bonds and their lengths (**C**,**D**). The *2Fo-Fc* electron density map of NBD-14204 (**E**) and NBD-14208 (**F**) contoured at 1σ.

To determine the crystal structures of HIV-1 gp120 core in complex with NBD-14204 or NBD-14208, we used a clade A/E 93TH057 HIV-1 gp120 core with His375 to Ser substitution [22,23], which enabled us to produce well-diffracting crystals consistently. The structures revealed that the compounds mimicked the binding of Phe43 of CD4 to gp120 and occupied the cavity in the CD4 binding site of gp120, as shown in previously reported structures of NBD compounds [4,6,9,24]. The trifluoro-methane group of NBD-14204 in region I (Figure 2A,B) contacted Thr257, Glu370, Ile371, and Trp427 with hydrophobic interaction. The nitrogen atom in the pyridine ring made a hydrogen bond to the carbonyl oxygen of Asn425. In addition, the pyrrole ring showed hydrophobic contact in region II with Trp427 (Figure 2C). Similarly, the 2-fluoro,1-chloro-derivatized phenyl ring of NBD-14208 in the region I made hydrophobic contacts with Thr257, Glu370, and Trp427 in the cavity (Figure 2C). Unlike NBD-14204, the nitrogen atom in the pyrrole ring of NBD-14208 made a hydrogen bond to the carbonyl oxygen of Asn425 (Figure 2D). Both structures showed that their amine groups in region III made hydrogen bonds to Asp368 (Figure 2C,D).

**Figure 3:**
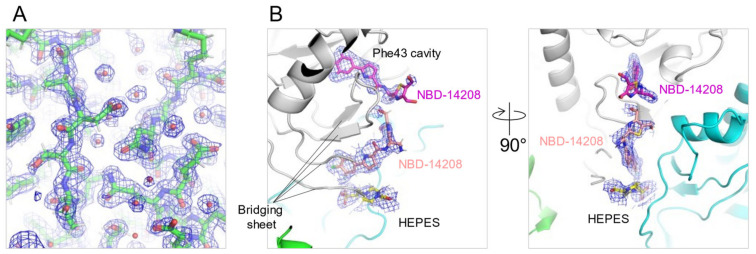
Electron density of NBD-14204- or NBD-14208-bound gp120 structures. (**A**) The *2Fo-Fc* electron density map of NBD-14204 bound gp120 contoured at 1σ. (**B**) Additional NBD-14208 compound was found under the bridging sheet region. Green and cyan molecules represent neighboring gp120s found in the crystal lattice.

However, the electron density of region III, particularly their thiazole groups, was poorly defined (Figure 2 E,F), even though the two structures were determined with relatively high resolutions of 1.9 Å and 2.1 Å for NBD-14204 and NBD-14208 (Figure 3A and Table 3), respectively, indicating that the thiazole rings in the region III are flexible and need to be further optimized to enable the region to make favorable interactions with neighboring residues. Interestingly, an additional NBD-14208 compound was found outside the Phe43 cavity, under the bridging sheet where NBD-14208 and a HEPES molecule were nestled in the space formed by neighboring gp120 molecules in the crystal lattice (Figure 3B).

## 3. Materials and Methods

### 3.1. Cells and Viruses

TZM-bl cells [25] were obtained through the NIH ARP. HEK293T/17 cells were purchased from ATCC. HIV-1 molecular clone expression vector pHXB2-env (X4) DNA was obtained through the NIH ARP [26]. The HIV-1 Env molecular clones of gp160 genes, which were used to produce the panel of clinical isolates of HIV-1 pseudoviruses, were obtained through the NIH ARP as follows: clones A (QH209.14M.ENV.A2 and QB726.70M.ENV.C4), AD, D, D/A, and C (QB099.391M.Env.B1) were obtained from J. Overbaugh [27,28,29]. The HIV-1 Env molecular clones panel of subtypes A/G and A/E were obtained from Drs. D. Ellenberger, B. Li, M. Callahan, and S. Butera [30]. The subtype B clones, QH0692 clone 42, PVO clone 4, and AC10.0.29 (SVPB13), were obtained from Drs. D. Montefiori and F. Gao [31]. The subtype B clone pRHPA4259 clone 7 (SVPB14), subtype C clone Du172 clone 17, ZM249M.PL1, and SVPC10 were obtained from Drs. B. H. Hahn, Y. Li J. F. Salazar-Gonzalez [32]. B clone pTHRO4156 clone 18 (SVPB15) was obtained from Drs. B. H. Hahn and D. L. Kothe. B clone 1058_11.B11.1550 was obtained from Drs. B. H. Hahn, B. F. Keele, and G. M. Shaw [33]. Subtype C clone Du422 clone 1 (SVPC5) was obtained from Drs. D. Montefiori, F. Gao, C. Williamson, and S. Abdool Karim. Clone C ZM53M.PB12 SVPC11 was obtained from Drs. C.A. Derdeyn and E. Hunter [34]. The HIV-1 Subtype C clone HIV-25925-2 clone 22 was obtained from Drs. R. Paranjape, S. Kulkarni, and D. Montefiori. The Env-pseudotyped genes of BG505.T332N, KNH1144, and B41 were kindly provided by J. P. Moore of the Weill Cornell Medical College, NY. The Env-deleted proviral backbone plasmid pSG3^Δenv^ DNA (from J. C. Kappes and X. Wu) [25,35] was obtained from the NIH ARP Division of AIDS, NIAID, NIH. MLV gag-pol-expressing vector pVPack-GP, Env-expressing vector pVPack-VSV-G, and a pFB-Luc vector were obtained from Stratagene (La Jolla, CA, USA).

### 3.2. Pseudovirus Preparation

Pseudoviruses capable of single-cycle infection were prepared as previously described [4,7]. Briefly, 8 × 10^6^ HEK293T/17 cells were transfected with the HIV-1 Env-deleted proviral backbone plasmid pSG3^Δenv^ and an HIV-1 Env-expression plasmid by using FuGENE HD (Promega, WI, USA). The control VSV-G pseudovirus was prepared by transfecting the cells with a combination of the Env-expressing plasmid pVPack-VSV-G, the MLV gag-pol-expressing plasmid pVPack-GP, the pFB-Luc plasmid, and FuGENE HD. Pseudovirus-containing supernatants were collected 2 days after transfection, filtered, titred, and stored in aliquots at −80 °C.

### 3.3. Measurement of Antiviral Activity

NBD-14204 and NBD-14208 were evaluated by infecting TZM-bl cells with pseudovirus lab-adapted HIV-1_HXB-2_ (CXCR4-tropic) in a single-cycle infection assay. Additionally, these compounds were evaluated against 25 HIV-1 pseudoviruses, pseudotyped with the Env from the clinical isolates panel. Briefly, TZM-bl cells were plated at 1 × 10^4^/well in a 96-well tissue culture plate and incubated overnight. The following day, aliquots of HIV-1 pseudovirus were pre-treated with the NBD compounds at graded concentrations for 30 min at room temperature and added to the cells. Following 3-day incubation, the cells were washed and lysed with 50 µL of lysis buffer (Promega, WI, USA). Twenty µL of the lysates were transferred to the wells of a white 96-well plate and mixed with the luciferase assay reagent (Promega, WI, USA). The luciferase activity was measured immediately with a Tecan Spark reader, and the percent of inhibition compared to the untreated positive control and the IC_50_ (the half-maximal inhibitory concentration) values were calculated using the GraphPad Prism software. The neutralization activity of the compounds against the control pseudovirus VSV-G was evaluated in U87-CD4-CXCR4 cells as previously described [13].

### 3.4. Evaluation of Cytotoxicity

The cytotoxicity of NBD-14204 and NBD-14208 was evaluated in parallel with the neutralization assay in TZM-bl cells using the colorimetric CellTiter 96^®^ AQueous One Solution Cell Proliferation Assay (MTS) (Promega, WI, USA) following the manufacturer’s instructions. Briefly, the cells were plated at 1 × 10^4^/well and cultured at 37 °C overnight. The following day, graded concentrations of the compounds were added to the cells and incubated for three days. Finally, the MTS reagent was added to the cells and incubated for 4 h at 37 °C. The absorbance was recorded at 490 nm. The percent of cytotoxicity and the CC_50_ (the concentration for 50% cytotoxicity) values were calculated as above.

### 3.5. Reverse Transcriptase Inhibition Assay

The activity of the NBD-14204 and NBD-14208 was evaluated against the HIV-1 Reverse Transcriptase (RT) by using the Colorimetric Reverse Transcriptase Assay (Roche) and following the manufacturer’s instructions. Nevirapine (NNRTI) and NBD-556 [36] were used as controls.

### 3.6. Crystallization, Data Collection, and Structural Refinement

Clade A/E 93TH057 gp120 core with H375S mutation was expressed in 293F cells, purified with a 17b-conjugated affinity column, and deglycosylated with Endoglycosidase H, as described previously [23]. The deglycosylated gp120 core was purified using HiLoad 16/600 Sperdex 200 pg size-exclusion chromatography column (GE Healthcare, NY, USA) with a running buffer containing 5 mM HEPES, pH 7.4, 150 mM NaCl. A molar excess of NBD-14204 or NBD-14208 in DMSO was added to the concentrated gp120 core to maintain the final concentrations of DMSO and gp120 of 5% and ~10 mg/mL, respectively. The NBD-14204 or NBD-14208/gp120 complexes were then screened for crystallization conditions using Hampton Research, Wizard Screen, and Precipitation Synergy Screen. The diffracting quality crystals of the complexes grew at 12–14% PEG3350, 5% isopropanol, 0.1M HEPES, 7.4. Crystals were flash frozen in the presence of cryoprotectant containing 30% glycerol, 14% PEG3350, 5% isopropanol, and 0.1M HEPES, 7.4 for data collection at the synchrotron beamline, SER-CAT ID22, at Advanced Photon Source (Lemont, IL, USA). The diffraction data were collected and processed using HKL2000 [37]. The structures were solved by molecular replacement using Phaser [38] with PDB ID 3TGT as a search model, built with Coot [39], and refined using Phenix [40]. Figures were generated by using Ligplot [41], PyMOL [42], and Chimera [42].

## 4. Conclusions

This report presented the antiviral activity of two different series of NBD-type small molecules against a diverse set of HIV-1 Env-pseudotyped clinical isolates and their crystal structures. The antiviral data indicated that the pyridine-containing compound, NBD-14204, showed about five-fold better antiviral activity than the phenyl-containing compound, NBD-14208. Furthermore, NBD-14208 appeared to be more cytotoxic than NBD-14204. However, in crystal structures, both compounds have some common interactions. For example, the primary amine group in region III of both compounds forms an H-bond (salt-bridge) with Asp368. Both the pyridine and phenyl rings also have similar hydrophobic interactions. However, one key difference is that the pyridine ‘N’ of NBD-14204 interacts with Asn425, whereas the pyrrole ‘N’ of NBD-14208 shows interaction with Asn425. These two inhibitors’ structural information and antiviral activity are expected to provide critical information to further design this series of inhibitors with improved efficacy and selectivity index.

## Figures and Tables

**Table 1:** Neutralization Activity of NBD Compounds against a selection of Env-pseudotyped HIV-1 clinical isolates.

			IC_50_ (µM) ^a^	
Subtype	ARP ^#^	ENVs	NBD-14204	NBD-14208
A	11895	QH209.14M.ENV.A2	0.27 ± 0.1	4.4 ± 0.1
A	11888	QB726.70M.ENV.C4	0.4 ± 0.2	2.87 ± 0.2
A		BG505-T332N	0.8 ± 0.04	4.7 ± 0.4
A		KNH1144	0.48 ± 0.05	3.6 ± 0.1
AD	11901	QA790.204I.ENV.A4	0.24 ± 0.01	3.4 ± 1
AE	11603	CRF01_AE clone 269	0.53 ± 0.04	5.7 ± 1.3
AG	11594	CRF02_AG clone 250	0.32 ± 0.01	1.87 ± 0.08
AG	11605	CRF02_AG Clone 278	0.53 ± 0.04	4.4 ± 0.16
B		B41	0.9 ± 0.1	4.2 ± 0.1
B	11018	QH0692, clone 42	0.48 ± 0.05	3.6 ± 0.1
B	11022	PVO, clone 4	0.5 ± 0.04	0.66 ± 0.03
B	11024	AC10.0.29 (SVPB13)	0.33 ± 0.03	2.3 ± 0.1
B	11036	pRHPA4259 clone7 (SVPB14)	0.56 ± 0.05	1.1 ± 0.2
B	11037	pTHRO4156 clone 18 (SVPB15)	0.29 ± 0.01	1.9 ± 0.04
B	11563	1058_11.B11.1550 ^b^	0.58 ± 0.02	3.7 ± 0.09
C	11307	Du172, clone 17	0.6 ± 0.15	3.7 ± 0.3
C	11308	Du422, clone 1	0.65 ± 0.03	3.8 ± 0.2
C	11312	ZM249M.PL1, SVPC10	0.46 ± 0.05	2.8 ± 0.3
C	11313	ZM53M.PB12, SVPC11	0.7 ± 0.09	3.8 ± 0.3
C	11507	HIV-25925-2, clone22	0.29 ± 0.01	1.85 ± 0.5
C	11908	QB099.391M.ENV.B1	0.3 ± 0.02	1.5 ± 0.08
D	11911	QA013.70I.ENV.H1	0.35 ± 0.1	1.7 ± 0.1
D	11912	QA013.70I.ENV.M12	0.24 ± 0.1	3.3 ± 0.5
D	11916	QD435.100M.ENV.B5	0.54 ± 0.06	1.7 ± 0.2
D/A	11526	MF535.WOM.ENV.C1	0.48 ± 0.07	2.8 ± 0.6
Mean ± SEM (µM): Overall (n = 25) SI			0.47 ± 0.03 310	3 ± 0.25 27.5
Subtype A (n = 4)			0.49 ± 0.11	3.9 ± 0.4
Subtype B (n = 7)			0.52 ± 0.08	2.5 ± 0.5
Subtype C (n = 6)			0.5 ± 0.07	2.9 ± 0.4
Subtype D (n = 3)			0.38 ± 0.09	2.2 ± 0.5
Subtype Rec (n = 5)			0.39 ± 0.07	3.4 ± 0.8
Toxicity CC_50_ (µM) ^a^			146 ± 3	82.5 ± 1.5

^a^ Reported IC_50_ and CC_50_ values represent the means ± standard deviations (n = 3). ^b^ CCR5/CXCR4-tropic virus all the rest are CCR5-tropic viruses. ^#^ARP, AIDS Reagent Program

**Table 2:** Antiviral Activity of NBD Compounds against HIV-1 Reverse Transcriptase, HIV-1_HXB2_ and Clinical Isolates (CI) and relative ratios.

Inhibitors	RT IC_50_ (μM) ^a^	HXB2		Clinical Isolates (CI)	
		IC_50_ (μM)	Ratio RT/HXB2	Mean IC_50_ (μM)	Ratio RT/CI
NBD-14204	8.3 ± 1.2	0.96 ± 0.1	8.6	0.47 ± 0.03	17.7
NBD-14208	5 ± 0.5	2.3 ± 0.1	2.2	3 ± 0.25	1.7
NBD-556	>300	-	-	-	-
Nevirapine	0.2	-	-	-	-

^a^ The reported IC_50_ values represent the means ± standard deviations (n = 2).

**Table 3:** Data collection and refinement statistics.

	NBD-14204:gp120	NBD-14208:gp120
Data collection		
Space group	P212121	P212121
Cell dimensions		
a, b, c (Å)	65.2, 67.6, 87.9	61.2, 66.9, 90.4
α, β, γ (°)	90, 90, 90	90.0, 90, 90
Resolution (Å)	50–1.90 (1.93–1.90) *	50–2.09 (2.18–2.09) *
Rsym	0.076 (0.82)	0.14 (0.56)
I/sI	13.5 (2.4)	14.5 (1.1)
Completeness (%)	92.2 (74.1)	94.2 (71.0)
Redundancy	4.3 (3.1)	6.4 (2.9)
**Refinement**		
Resolution (Å)	36.4–1.94	36.4–2.09
No. reflections	24,631	21,346
Rwork/Rfree	18.2/21.6	24.2/29.7
No. atoms		
Protein	5308	5278
Ligand/ion	254	370
Water	277	49
B-factors (Å2)		
Protein	26.4	59.6
Ligand/ion	40.8	76.8
Water	32.9	59.5
R.m.s. deviations		
Bond lengths (Å)	0.008	0.011
Bond angles (°)	0.767	0.760
Ramachandran plot (%)		
Favored	97.3	96.65
Allowed	2.70	3.35
Outliers	0.00	0.00
PDB ID	8F9Z	8FA0

* Values in parentheses are for the highest-resolution shell.

## Data Availability

Not applicable.
